# Nucleic Acid Target Sensing Using a Vibrating Sharp-Tip Capillary and Digital Droplet Loop-Mediated Isothermal Amplification (ddLAMP)

**DOI:** 10.3390/s24134266

**Published:** 2024-06-30

**Authors:** Bethany J. Fike, Kathrine Curtin, Peng Li

**Affiliations:** 1 C. Eugene Bennett Department of Chemistry, West Virginia University, Morgantown, WV 26506, USA; bf0044@mix.wvu.edu (B.J.F.); kc0027@mix.wvu.edu (K.C.); 2 Department of Mechanical and Aerospace Engineering, West Virginia University, Morgantown, WV 26506, USA

**Keywords:** nucleic acid testing, digital detection, LAMP, DNA fragmentation, POC

## Abstract

Nucleic acid tests are key tools for the detection and diagnosis of many diseases. In many cases, the amplification of the nucleic acids is required to reach a detectable level. To make nucleic acid amplification tests more accessible to a point-of-care (POC) setting, isothermal amplification can be performed with a simple heating source. Although these tests are being performed in bulk reactions, the quantification is not as accurate as it would be with digital amplification. Here, we introduce the use of the vibrating sharp-tip capillary for a simple and portable system for tunable on-demand droplet generation. Because of the large range of droplet sizes possible and the tunability of the vibrating sharp-tip capillary, a high dynamic range (~2 to 6000 copies/µL) digital droplet loop-mediated isothermal amplification (ddLAMP) system has been developed. It was also noted that by changing the type of capillary on the vibrating sharp-tip capillary, the same mechanism can be used for simple and portable DNA fragmentation. With the incorporation of these elements, the present work paves the way for achieving digital nucleic acid tests in a POC setting with limited resources.

## 1. Introduction

Nucleic acid amplification tests (NAATs) are key tools for the screening, risk assessment, diagnosis, therapy selection, prognosis, and monitoring of many diseases [1]. With the recent COVID-19 pandemic, the need for rapid and accurate nucleic acid testing has been shown in recent years [2,3,4]. While conventional real-time polymerase chain reaction (PCR) [5] methods have demonstrated tremendous success for qualitative analysis as well as semi-quantitative analysis, achieving the absolute quantification of target sequences is challenging. The impact of sample matrices and PCR inhibitors often undermines the reliability of copy number quantification. To overcome this limitation, digital PCR was developed, in which the bulk volume is divided into many smaller portions. Each of these portions containing either 0 or 1 copy of the target sequence is then amplified and read individually to determine the “negative” and “positive” portions [6,7,8]. Absolute quantification is then achieved based on the Poisson distribution. To date, digital PCR has been used in diverse applications, including pathogen detection, rare sequence detection, and liquid biopsy.

Despite the tremendous success in laboratory-based tests, developing point-of-care (POC) digital PCR technology has been challenging. To simplify the amplification process and eliminate the need for a thermal cycler, many isothermal amplification methods have been used for digital amplification. Notably, loop-mediated isothermal amplification (LAMP) [9] is the most widely performed reaction due to the high specificity and sensitivity of using loop structures to encourage the amplification of the target. To date, many digital LAMP (dLAMP) methods have been reported depending on different mechanisms for achieving sample compartmentalization. The first class of methods utilizes pre-formed or prefabricated structures for distributing bulk samples into small volumes. The use of gel arrays with LAMP reagents embedded inside for amplification has been reported as a possible method for dLAMP [10,11,12,13]. Similarly, dLAMP has been performed in commercially available membranes using the pores as a microwell array [14,15,16]. In addition, slipchip technology [17,18,19,20,21,22,23] and self-digitizing microfluidic chips [24,25,26] have been utilized to integrate additional fluid operations to distribute the bulk solution for dLAMP. In addition to these microwell-based dLAMP methods, water-in-oil emulsions (droplets) have also been used for dLAMP due to their advantage of generating a much larger number of individual amplifications with lower cost, termed droplet digital LAMP (ddLAMP). Microfluidic flow focusing and T-junction are the most used methods for generating monodisperse droplets for ddLAMP [27,28,29,30]. Although microfluidic-based methods work well for droplet generation, they often require multiple fluid pumps and microfabricated devices, making them difficult to use in POC systems. Non-microfluidic strategies, including centrifugal force-based [31,32,33,34,35], inkjet printing [36,37,38,39], and electrowetting methods [40], have also been reported to simplify the droplet generation process. However, sophisticated external equipment is still required for operation.

In recent years, droplet generation methods that are simple and portable have been reported. Yuan and colleagues reported a ddLAMP method utilizing a hand-powered droplet generator [41], in which manual syringe-based suction pumping is used to form droplets in a microfluidic chip. The droplets are collected in a container and heated with a water bath, and then they are transferred to a well plate for fluorescence imaging. The authors reported a limit of detection of 10 copies per microliter with the system that can be used in POC; however, the size of the droplets cannot be tuned, so only one average droplet size is possible. Similarly, Wu and colleagues reported a ddLAMP method utilizing microfluidic step-emulsion of droplets through the manual pumping of a syringe [42], in which the bacterial DNA targets were distributed into a chip, amplified using a water bath, and underwent fluorescence imaging for duplex detection. The authors reported a limit of detection of 19.8 copies per microliter. Mao and colleagues reported an open droplet array consisting of hydrophilic and hydrophobic patterning, which was sealed with mineral oil. The chip was then incubated, and smartphone-based fluorescent imaging and app-based analysis were performed [43]. The authors reported a limit of detection of 1 copy of lambda DNA per microliter; however, the method is limited by fixed dimensions of the pattern. Although these simplified droplet generation methods are more POC-friendly, the performance of these methods in terms of throughput, monodispersity, and droplet size flexibility is compromised due to the lack of sophisticated control equipment, which leads to reduced analytical performance for digital assays.

Recently, our lab reported a simple and compact droplet generation method based on a vibrating sharp-tip capillary [44]. This system eliminates the need for external pumps and achieves on-demand droplet generation with a portable signal generation, which shows great potential for POC digital nucleic acid tests. In this work, we combine the vibrating sharp-tip capillary droplet generation with LAMP to achieve a simple digital droplet assay for detecting bacteria pathogen sequences. We generated a large range of stable droplet sizes (~0.7 pL to 22 nL) with a single device. We optimized the conditions for the digital LAMP reactions and employed a simple DNA fragmentation method to ensure uniform sample distribution throughout the experiments. Finally, we detected common waterborne pathogens, Vibrio cholerae and Shigella sonnei, with a linear dynamic range of approximately 2 to 6000 copies/μL. This method eliminates the need for bulky equipment for generating monodisperse droplets and a thermal cycler, which paves the way for a portable and high dynamic range ddLAMP system.

## 2. Materials and Methods

### 2.1. Reservoir Preparation

Glass slides (25 × 76 × 1 mm, Fisher Scientific, Pittsburgh, PA, USA) were cut down to the same approximate dimensions as glass cover slides (24 × 60 mm, VWR, Radnor, PA, USA). The cut slides were placed through air plasma treatment and then placed into a 0.1 M NaOH solution for 1 h. The slides were then transferred to a hydrophobic coating solution consisting of Octadecyltrimethoxysilane (OTS) (TCI, Portland, OR, USA) (300 μL) in Toluene (60 mL) for 2 h. The slides were then left to air-dry propped upright in a fume hood overnight. The glass slides were transferred to Petri dishes and placed in a 60 °C oven. Polydimethylsiloxane (PDMS) solution (SYLGARD™ 184 Silicone Elastomer Kit, DOW, Midland, MI, USA) was prepared 10:1 (polymer to curing agent) and degassed until all air bubbles were removed. The PDMS solution was then poured into Petri dishes with silicon wafers affixed to the bottom. The PDMS was then baked in the same oven for 2 h. The previously treated glass slides and PDMS were removed from the oven. PDMS was allowed to cool to room temperature, while glass slides were placed into a methanol soak on a shaker. While PDMS slabs were cut to form individual reservoirs, the glass slides were shaken. Once the PDMS slabs were cut, the glass slides were removed from the methanol, rinsed with water, and dried with pressurized air. Scotch tape was used to remove any debris from both the PDMS reservoirs and the glass slides. The PDMS and glass slides were air-plasma-treated and then bonded together. The formed reservoirs were placed back into the oven to finish curing for future use.

### 2.2. Vibrating Sharp-Tip Capillary Device Preparation

A piezoelectric transducer (4.6 kHz, 27 mm, Murata, Thief River Falls, MN, USA) was attached to a glass cover slide using UV curing glue (Norland Optical Adhesive, Norland, Cranbury, NJ, USA). A weight was used to ensure firm contact between the transducer and slide as well as remove any trapped air bubbles. The glue was cured under a UV lamp for 10 min. A pre-pulled glass pipette capillary (TIP20TW1, World Precision Instruments, Sarasota, FL, USA) with a 20 μm tip was attached at an angle to the prepared device base using glass glue (Locktite^®^, Henkel, Rocky Hill, CT, USA). A piece of scotch tape was used to affix the device to the storage setup, and the device was labeled with the size of the tip. Each device was then stored by securing the device inside a sealed Petri dish.

The vibrating sharp-tip capillary device for DNA fragmentation was prepared in a similar manner, except for using a PUL-1000 Four-Step Micropipette glass capillary Puller (World Precision Instruments, Sarasota, FL, USA) to pull tips (preset program 01) in place of the commercial glass capillary.

### 2.3. Tubing Preparation

Lengths of large (0.86 mm I.D. × 1.5 mm O.D.) LDPE tubing (BB31695-PE/6, Scientific Commodities, Inc., Lake Havasu, AZ, USA), approximately 15.2 cm, and 30-gauge PTFE tubing (SWTT-30-C, Component Supply, Sparta, TN, USA), approximately 3.8 cm, were cut using a razor blade. Each piece of tubing was cleaned using either ethanol or isopropanol. The smaller tubing was inserted into the larger tubing, with approximately 19 mm remaining from the end. The tubing connection was sealed using 5-Minute Epoxy (Devcon^®^, ITW Performance Polymers, Danvers, MA, USA), and the tubing was hung from the benchtop until dry.

### 2.4. DNA Fragmentation

The stock solution of DNA was diluted by 10 times to 50 μL in a 200 μL microtube. The DNA fragmentation vibrating sharp-tip capillary was checked under an Olympus microscope to ensure the cut tip outer diameter was between 30 and 50 μm in diameter. The device was attached to a stand and aligned so that the tip of the device was inserted into the DNA sample. The generator leads were attached to the leads on the piezoelectric transducer, and the signal ran for 30 s at 95.0 kHz and 10 Vpp. The DNA was then vortexed for a few seconds to ensure uniform mixing throughout the sample.

### 2.5. ddLAMP Reaction

A primer mix was prepared at 10× concentration in the following amounts for 100 μL of mix from 100 μM stock solutions: 2 μL F3, 2 μL B3, 4 μL Loop F, 4 μL Loop B, 16 μL FIP, 16 μL BIP, and 56 uL nuclease-free water. Primer sequences used are listed in the Supporting Information (Table S1) [45] and were purchased from IDT (Integrated DNA Technologies, Inc., Coralville, IA, USA). LAMP WarmStart^®^ Master mix (E1700, New England Biolabs^®^, Inc., Ipswich, MA, USA), 10× Primer mix, 20× EvaGreen^®^ Dye in water (#31000, Biotium, Fremont, CA, USA), DNA, and Ambion™ Nuclease-Free Water (AM9938, Invitrogen, Austin, TX, USA) were removed from freezer/refrigerator and warmed to room temperature. The vibrating sharp-tip capillary devices for droplet generation were checked under a microscope to ensure the tips were free from chips, breakages, and blockages. Devices were then cleaned using a small Petri dish of deionized water in a sonicator and a syringe with tubing attached to the device capillary, using the combination of sonication and suction to clean and remove any potential blockages. These devices were then placed in the oven to remove excess water inside the capillary while the LAMP reaction mixture was prepared. Fragmented DNA was diluted using nuclease-free water. The ddLAMP reaction solution was prepared in low-binding 1.7 mL PCR microtubes using the following amounts for the final volume of 100 μL solution: 50 μL 2× WarmStart^®^ LAMP master mix; 10 μL 10× Primer mix; 5 μL EvaGreen^®^ dye; 4 μL DNA (ATCC, Manassas, VA, USA) or water for NTC; and 31 μL nuclease-free water. Previously prepared reservoirs were placed onto a removable thermal cycler platform and labeled using an ultrafine-tip dry-erase marker. Additionally, an extra separate reservoir was prepared to test droplet generation parameters. All reservoirs were filled with 7% (*w*/*v*) Gransurf 90 (Grant Industries, Elmwood Park, NJ, USA) in mineral oil (Sigma Aldrich, St. Louis, MO, USA) (3.50 g in 50 mL) with a flat surface corresponding to the top of the reservoir. Syringes were prepared by attaching a 27-gauge needle, gently removing the cap, and introducing approximately 300 μL of air to the syringe. The LAMP reaction solution was mixed using a pipette before being loaded into the prepared syringe through the needle by suction. The loaded syringe was attached to the prepared tubing, which was then attached to the vibrating sharp-tip capillary device for droplet generation. The device was attached to the stand with the capillary parallel to the side of the stand while allowing free movement. The LAMP reaction solution was manually loaded through the tubing into the capillary until a few drops were released from the tip. The syringe was then removed from the tubing, and the tubing was taped in place. The signal generator and amplifier were connected to the leads attached to the piezoelectric transducer on the device. The running frequency and amplitude for the device were adjusted under a microscope until the liquid flow was a steady continuous flow. The droplet generation was then tested by switching the acoustic generator from continuous flow to pulsed signal. After droplet generation for four replicants, the droplets were covered with an overturned Petri dish, and a piece of paper to ensure no dust or light entered the reaction reservoirs while the droplets settled into a monolayer (approximately 15 min). The reaction reservoirs were loaded into a thermal cycler where LAMP occurred at 65 °C for 1 h before cooling to 4 °C until ready for imaging. Each droplet field was imaged under a microscope with both bright field and fluorescence images at each position in an “S” pattern until all droplets were imaged.

### 2.6. Image Analysis and DNA Counting

Two methods of image analysis were performed using computer software ImageJ (Version 1.52a, U.S. National Institutes of Health, Bethesda, MD, USA), CellProfiler™ (Broad Institute, Cambridge, MA, USA), and “Define the Rain”. With the first analysis method, each image set was analyzed manually. The average droplet diameter of the droplet field was determined with the use of ImageJ. The average droplet diameter was then converted into the average droplet volume. Each bright field image was then analyzed to determine the total number of droplets in the image as well as the full droplet field. Next, each fluorescence image was analyzed to determine the number of positive droplets in the droplet field. The results for the total number of droplets, the number of positive droplets, and the average droplet volume were collected and used to calculate the copies of DNA targets for the individual droplet reservoirs.

In contrast to the manual method, an automated analysis method was also developed. A single bright field image was analyzed using ImageJ to determine the average droplet diameter, and the average droplet volume was calculated in the same manner as the manual analysis method. All the fluorescence images for a droplet field were uploaded into the CellProfiler™ software (Version 4.2.4). The software then analyzed the images according to the input parameters. When the software analysis was completed, the resulting fluorescence information was reported as a percentage of the maximum fluorescence intensity possible. As a result, the fluorescence intensity percentages were converted to actual fluorescence intensity values, and these fluorescence intensity values were saved in a “.csv” format. The saved “.csv” file was uploaded to the “Define the Rain” website. The software converted the uploaded data into a histogram and then sorted the values into “negative” and “positive” signals. The total number of reported droplets, the number of reported positive droplets, and the average droplet volume were recorded, and the number of copies of DNA targets for the individual droplet reservoirs was calculated.

With both methods, calculating DNA copy number counting was performed using the following equation:

(1) $$C=\frac{-\left(\ln\left(1-\frac{p}{n}\right)\right)}{V},$$

where *C* is the calculated DNA copy number, *p* is the number of positive droplets, *n* is the total number of droplets, and *V* is the average droplet volume.

## 3. Results and Discussion

### 3.1. Droplet Generation Using Vibrating Sharp-Tip Capillary

**Figure 1:**
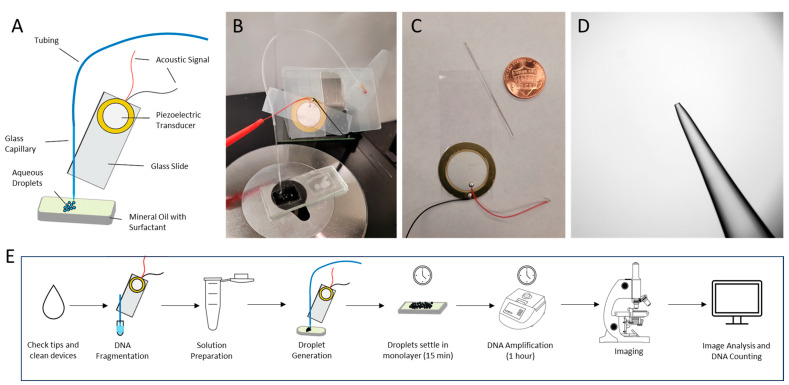
The vibrating sharp-tip capillary for droplet generation: (**A**) schematic drawing of setup; (**B**) real image of setup; (**C**) vibrating sharp-tip capillary device with penny for scale; (**D**) tip of capillary; (**E**) schematic workflow of ddLAMP.

**Figure 2:**
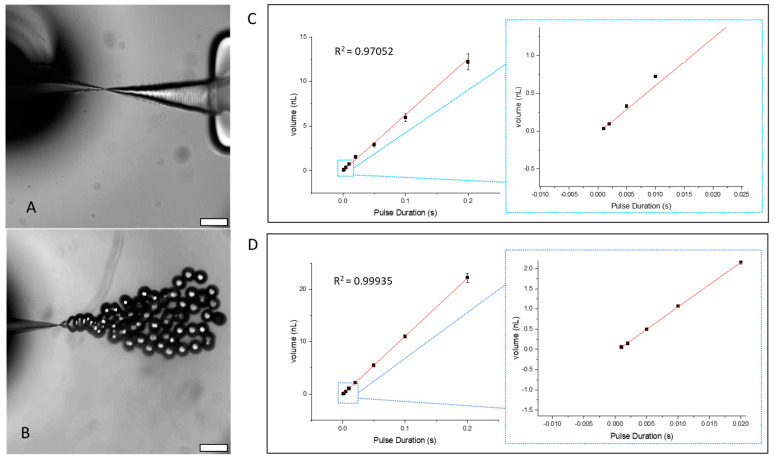
Mechanism of droplet generation with the vibrating sharp-tip capillary using continuous flow (**A**) and pulsed signal (**B**) modes (scale bar-400 μm). Linear relationship of droplet volume vs. pulse signal duration using a 20 µm tip with an expanded view of low pulse durations at 3.5 Vpp (**C**) and 4.0 Vpp (**D**).

In this work, droplet generation was achieved using a vibrating sharp-tip capillary, as shown in Figure 1A. The system was composed of a glass capillary with a pulled tip, a glass slide, and a piezoelectric transducer. When RF signals were applied to the piezoelectric transducer, oscillating vibration occurring at the tip drove the liquid from the capillary (Figure 2A, Video S1). Due to the strong acoustic streaming induced by the vibrating sharp tip, the meniscus between the aqueous and oil phase was compressed to a “triangle” shape, which was sustained by the pumping pressure of the vibrating capillary. When the vibration was stopped, the Laplace pressure pinched off the liquid at the tip of the capillary. Based on this mechanism, applying pulsed RF signals led to the generation of highly monodispersed droplets (Figure 2B, Video S2). In this work, the pulsed signal was achieved through amplitude modulation (AM) with a square wave. Due to the simple mechanism of the vibrating sharp-tip capillary, only a low-cost signal generator is needed for droplet generation. In addition, the low power consumption of the device ensures that even a battery-powered acoustic generator can be used, which allows for a compact and portable method of droplet generation. Another benefit of this droplet generation mechanism is that both droplet number and droplet size can be controlled. Generating various droplet sizes is particularly beneficial to digital PCR assays as the large range of droplet sizes creates a larger dynamic range than would be possible with a single droplet volume.

By working under continuous flow, the device was observed under a microscope and tuned to its running frequency as each device operated at slightly different parameters. Under the continuous mode, the activation frequency and input voltage were adjusted within the range of 93.0 to 100.0 kHz and 2 to 8 Vpp considering activation frequency and input voltage, respectively. These parameters were adjusted to ensure a strong and steady continuous flow of liquid from the tip of the capillary. Once the operating frequency and voltage were determined, the acoustic signal was then switched to a pulsed signal for individual droplets to form. The pulsed signal was achieved through amplitude modulation (AM) with a square wave. As a result, when the acoustic signal was “on”, the pumping of the liquid occurred from the tip of the device. However, when the acoustic signal was “off”, the pumping of the liquid stopped, and the liquid flow pinches closed, allowing for individual droplets to form. Due to the simple mechanism of the vibrating sharp-tip capillary, only an acoustic generator is needed for the pumping mechanism. In addition, the low power consumption of the device ensures that even a battery-powered acoustic generator can be used, which allows for a compact and portable method of droplet generation. This compact and portable device, in conjunction with an isothermal amplification method (LAMP), would increase the accessibility of digital amplification detection of nucleic acids in low-resource areas.

To tune the droplet size with the vibrating sharp-tip capillary, the input voltage and AM frequency can be adjusted. Under a constant operating frequency and input voltage, the AM frequency can be changed to control the size of the droplets, as shown in Video S3. With a low AM frequency (5 Hz) and long pulse duration (0.2 s), the formed droplets are large in volume. With a high AM frequency (1000 Hz) and short pulse duration (0.001 s), the formed droplets are small in volume. The AM frequency also coordinates the number of droplets formed per second. The relationship between the average droplet volume and the length of the pulsed signal duration is linear, as shown in the graphs of Figure 2C,D, down to a pulsed signal duration of 0.001 s. At high AM frequencies (greater than 1000 Hz), the trend shifts from a linear relationship to a plateau, although the vibrating sharp-tip capillary can still perform at these higher pulse frequencies, as shown in Tables S2 and S3. In addition to changing the pulsed signal duration, the droplet volume can be changed by adjusting the input voltage. When the voltage increased, the droplet volume also increased due to the increase in the pumping flow rate. This is evident by comparing Figure 2C, in which the input voltage is 3.5 Vpp, and Figure 2D, in which the input voltage is 4.0 Vpp. The higher input voltage (Figure 2D) shows larger droplet volumes than the lower input voltage (Figure 2C). By changing these two parameters, a large range of droplet volumes is possible. With a single device, we generated stable droplets of approximately 22 nL (~350 μm in diameter) down to approximately 0.7 pL (~11 μm in diameter).

### 3.2. Optimization of ddLAMP

**Figure 3:**
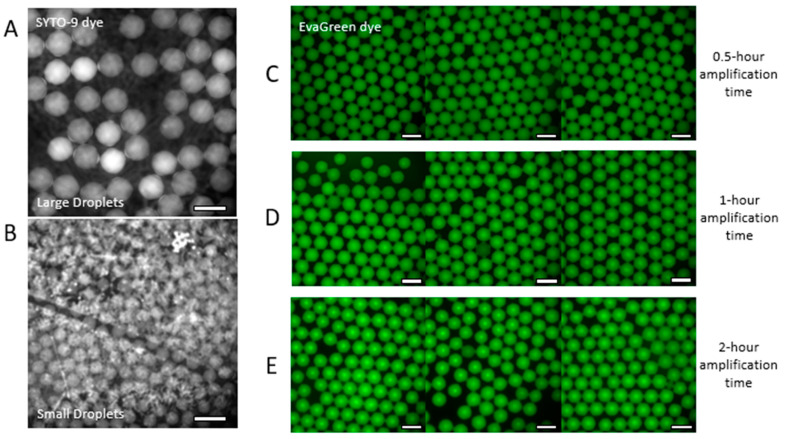
Optimization of ddLAMP conditions. Interference of fluorescence signal with using DMSO-based SYTO-9 dye (**A**,**B**). Effect of amplification time duration on fluorescence signal (**C**–**E**) under same image fluorescence scale. (Scale bar—200 μm).

Using isothermal nucleic acid amplification would increase accessibility to low-resource areas. LAMP was chosen as the isothermal amplification method as it is often used as the “gold standard” for isothermal nucleic acid amplification. Throughout testing, the WarmStart^®^ LAMP kit from New England Biolabs was used. As this kit is typically used for bulk LAMP reactions, optimizations were needed for the ddLAMP reaction. The first optimization necessary was the fluorescent dye as the clear difference in fluorescence intensity is key in identifying negative and positive droplets. With preliminary tests, the SYTO-9 fluorescent dye provided in the WarmStart^®^ LAMP kit and a Listeria monocytogenes bacterial DNA genetic target were used. Because the solvent for the dye is dimethyl sulfoxide (DMSO), there were issues with using this dye. Specifically, since DMSO is an organic solvent, the SYTO-9 dye could leach out of the droplets through the mineral oil and surfactant mixture to adhere to the organically based hydrophobic coating. This adhesion caused optical interference during fluorescent imaging. This phenomenon, shown with larger droplets in Figure 3A, is more prevalent in the smaller droplets, as shown in Figure 3B. Because of the interference from the SYTO-9 dye, it was determined that a water-based fluorescent dye would be necessary. EvaGreen^®^ dye, which is a water-based fluorescent dye, was chosen for use with the ddLAMP protocol.

In addition to the fluorescent dye, the optimization of the optimal amplification time was also necessary. In contrast to bulk detection, with digital detection using droplet microfluidics, the volumes are much smaller, and the amount of reagents is limited. Also, each droplet contains a single DNA target, indicating that the exponential amplification will take time to reach a detectable level. For the digital detection of amplified nucleic acids, the fluorescence of each individual droplet is detected, and these droplets are then sorted into “negative” and “positive” groups. The key to sorting these droplets is to ensure that amplification reaches its endpoint. For these optimization tests, amplification times of 30 min, 1 h, and 2 h were used with the same DNA concentration as well as approximately the same droplet size (~145 μm in diameter). Although the recommended protocol for the LAMP kit used an amplification time of 30 min, there were inconsistencies in the percentage of positive droplets detected, due to the differences in the amplification of each droplet. Additionally, the average fluorescence intensity values of “negative” to “positive” droplets were 1754 ± 126 and 2189 ± 152, respectively. The 1 h and 2 h amplification times were also tested under the same conditions. For the 1 h amplification time, the average fluorescence intensity values for “negative” and “positive” droplets were 2199 ± 130 and 2921 ± 229, respectively, while the average fluorescence intensity values of the 2 h amplification time for “negative” and “positive” droplets were 2198 ± 221 and 3158 ± 236, respectively. The results of the fluorescence intensity tests are depicted in Figure S1. Because the contrast levels of fluorescence intensity for “negative” and “positive” droplets of both the 1 h and 2 h amplification times were similar, it was determined that a 1 h amplification time is sufficient for endpoint fluorescence measurements of the individual droplets. For all further testing, 1 h amplification time was used for the ddLAMP protocol.

### 3.3. DNA Fragmentation

During the droplet generation process, genomic DNA strands could settle in the capillary, leading to an increased concentration at the tip region for samples with higher DNA concentrations. This phenomenon affects the uniformity of the amplification results across different droplets. For example, the percentage of positive droplets was lower in the droplets that were generated later in the same experiment. To mitigate this issue, we implemented DNA fragmentation before loading the sample into the vibrating capillary for droplet generation, which would allow for less settling of the DNA as well as the prevention of the blocking of the tip of the capillary. Currently, the two most common methods of DNA fragmentation are enzymatic-based and sonication-based fragmentation. With enzymatic-based DNA fragmentation, the enzyme breaks the DNA sequence based on its specific activity, but purification and cleanup of the new ends of the DNA sequence are required for further use, making it not as accessible to low-resource areas [46]. In contrast, with sonication-based DNA fragmentation, the acoustic waves introduce small gas bubbles into the system, which break and cause the DNA to fragment without the need for cleanup, but these instruments are large, making them inaccessible [47]. Here, we utilized a vibrating sharp tip, the same setup as the droplet generation device, to achieve DNA fragmentation [48]. This method allows us to achieve DNA fragmentation without adding additional equipment or procedures, thereby maintaining the low resource requirement for the overall workflow.

**Figure 4:**
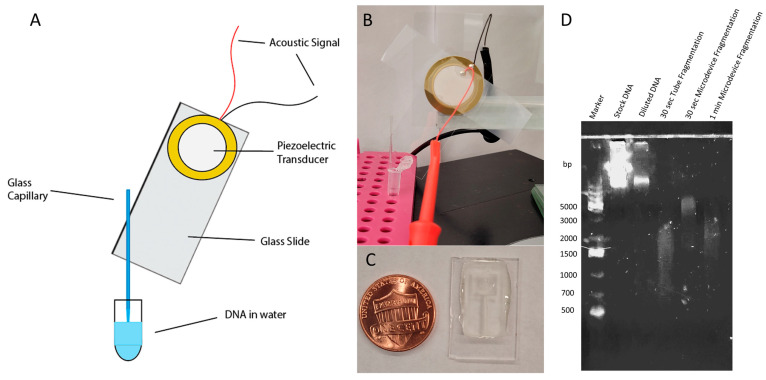
The vibrating sharp-tip capillary for DNA Fragmentation: (**A**) a schematic drawing of the device; (**B**) a real image of the device; (**C**) 3D fragmentation microdevice used; (**D**) gel electrophoresis results of fragmentation.

The setup of the DNA fragmentation device is shown in Figure 4A,B. The capillaries used for the DNA fragmentation device were pulled using a PUL-1000 Four-Step Micropipette Puller to form a tip with an outer diameter of approximately 35 μm. Input parameters of 95.0 kHz activation frequency and a voltage of 10 Vpp were used to break apart the DNA. When the RF signal was applied to the DNA fragmentation device, the vibration of the sharp tip of the capillary caused strong streaming of the liquid from the single point. This streaming caused fast and strong shearing of the DNA into smaller fragments. Furthermore, the volume of the DNA sample required was only approximately 50 μL, which is suitable for processing low-volume samples. DNA fragmentation with the vibrating sharp-tip capillary was tested using human male genomic DNA. Three 50 μL DNA samples contained in 200 μL microtubes were tested for fragmentation. The first sample was fragmented using the vibrating sharp-tip capillary device with the tip submerged in the 50 μL sample, and acoustics were applied for 30 s for fragmentation to occur. The second DNA sample was transferred from the microtube into a 3D-printed microdevice (10 mm × 18 mm × 2 mm), shown in Figure 4C, consisting of an open well (5 mm × 6 mm × 1 mm) and an insertion channel for the capillary (radius of 0.6 mm for 7 mm, followed by a 5° slope decrease for 3 mm). The acoustics were then applied to the capillary for 30 s for fragmentation to occur. The third DNA sample was also fragmented within the 3D-printed microdevice but with acoustics applied for 1 min. The fragmentation patterns of each of the DNA samples were monitored by agarose gel electrophoresis, as shown in Figure 4D. For the gel electrophoresis, three reference solutions were also prepared, which contained the reference DNA ladder, the pure unfragmented DNA stock, and the diluted unfragmented DNA solution. It was found that the 30 s fragmentation in the microtube produced a range of DNA fragments from approximately 600 to 3000 base pairs. In comparison, the 30 s fragmentation in the 3D microdevice produced a range of DNA fragments from approximately 1500 to 5000 base pairs. In addition, the 1 min fragmentation in the 3D microdevice produced fragments with a range of approximately 1000 to 4000 base pairs. Based on these results, the vibrating sharp-tip capillary produced the broadest range of DNA fragments in 30 s within the microtube. As a result, this setup was used for all further DNA fragmentation required in the ddLAMP protocol.

### 3.4. Automated Image Analysis

**Figure 5:**
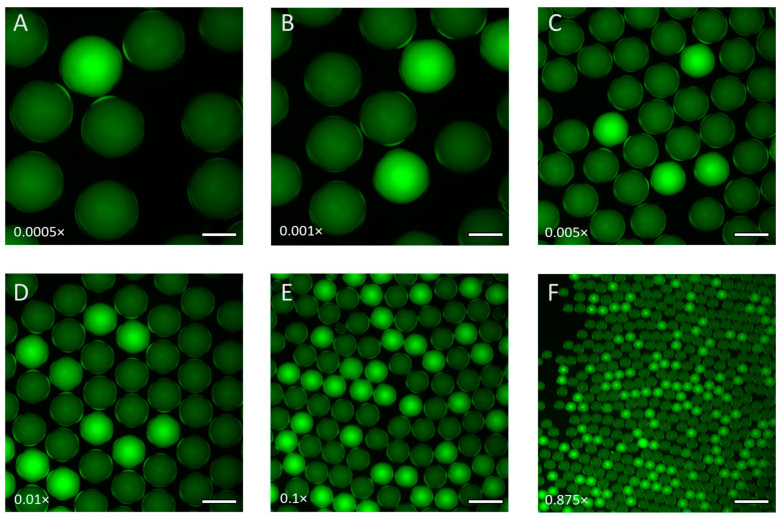
Visual representation of ddLAMP at six different concentrations and droplet sizes using Vibrio cholerae as the target: (**A**) 0.0005× with ~370 µm diameter; (**B**) 0.001× with ~325 µm diameter; (**C**) 0.005× with ~190 µm diameter; (**D**) 0.01× with ~195 µm diameter; (**E**) 0.1× with ~120 µm diameter; (**F**) 0.875× with ~65 µm diameter. (Scale bar-200 μm).

To improve the image processing time, two image analysis methods, described in length in the Section 2, were investigated to automate the image processing. First, the image processing was performed manually, as a baseline, to determine the DNA copy numbers for the sample as well as the amount of time necessary. In brief, each of the bright field images was used to determine the average size of the droplets as well as the total number of droplets in the sample reservoir. Each of the fluorescence images was then analyzed for the number of positive droplets contained in the sample reservoir identifying droplets, showing an increased fluorescence intensity compared to the dark, negative droplets. The DNA copy number was calculated using the collected values. As the contrast between the fluorescence of the negative and positive droplets is clear, as shown in Figure 5, visual analysis is simple to achieve in a range of droplet sizes down to approximately 65 μm in diameter. However, manual image processing is lengthy, potentially taking hours to complete. In contrast, a fully automated image analysis method was also investigated by combining two commercial software platforms, CellProfiler™ [49] and “Define the Rain” [50], as outlined in Figure S2. In brief, all the fluorescence images for a sample reservoir were uploaded into CellProfiler™, and the individual fluorescent readings of each droplet were recorded according to the input parameters. These fluorescence intensity values were uploaded to the “Define the Rain” website. The website converts the input data into a histogram and then determines the threshold cutoff between negative and positive. The average droplet volume was determined through bright field analysis using ImageJ software. Using the collected values, the DNA copy number could be calculated, and with the automated image analysis, the processing time was reduced to under 15 min per sample reservoir. A comparison of the DNA counts collected from the two different analysis methods revealed that both methods yielded similar results, indicating that both methods are viable methods of DNA copy number counting from fluorescence images in instances where the difference in fluorescence intensity is clear.

### 3.5. Quantification Performance of ddLAMP

**Figure 6:**
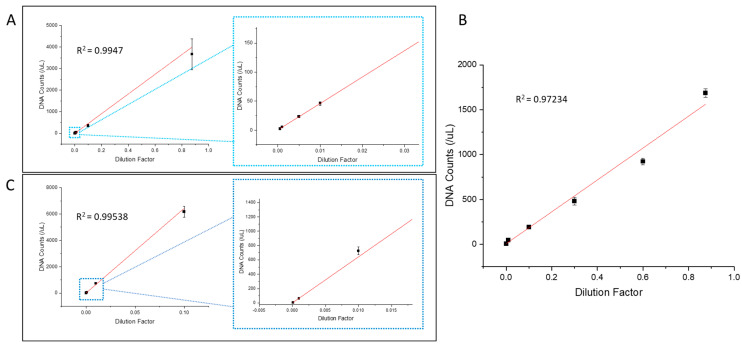
The dynamic range of two bacterial strains: (**A**) Vibrio cholerae with an expanded view of low concentrations; (**B**) Vibrio cholerae with additional concentrations; (**C**) Shigella sonnei.

Finally, we performed the quantification performance and dynamic range of the ddLAMP system, using the waterborne pathogens Vibrio cholerae and Shigella sonnei. For both bacteria, a DNA genetic target was chosen to be common among all strain types. By using the range of droplet volumes possible, a large dynamic range is possible using ddLAMP. For the Vibrio cholerae target, six different concentrations (dilutions) were tested (0.0005×, 0.001×, 0.005×, 0.01×, 0.1×, and 0.875×) with four replicants for each concentration. The size of the droplets formed was determined based on an estimation of the concentration and the droplet size required to be within the detection range. For example, with the lowest concentration (0.0005×), approximately 500 large droplets with a diameter of approximately 370 μm (~26 nL) were generated and analyzed for determination of the number of DNA copy targets detected in the individual reservoirs. For the highest DNA concentration (0.875×), approximately 9000 droplets with a diameter of approximately 65 μm (~120 pL) were generated and analyzed for the determination of the number of DNA copy targets detected. Based on the six concentrations and automated image analysis, the Vibrio cholerae target had a large linear dynamic range of approximately 2–3700 copies/µL, as shown in Figure 6A, with an R^2^ value of 0.9947. The six concentrations yielded average DNA copy number counts of 2, 6, 23, 46, 360, and 3664 copies/μL, respectively, from lowest to highest concentration (reported in Table S4). In addition, the relationship between the calculated DNA copy number counts and the dilution factor is linear, which is visible in the overall graph as well as the expanded view of the lower concentrations. For comparison, the graph of the manual image analysis is shown in Figure S3, with an R^2^ value of 0.9765. In addition, the two analysis methods are plotted together in Figure S4 for a direct comparison. Additional concentrations of the Vibrio cholerae DNA sample were also tested, which also resulted in a linear curve, as shown in Figure 6B.

The dynamic range was also tested using Shigella sonnei as the genetic target. For this target, only four concentrations (dilutions) were used: 0.0001×, 0.001×, 0.01×, and 0.1×. The testing of the concentrations for Shigella sonnei was performed in the same way as with the Vibrio cholerae, with four replicants for each of the concentrations. For the lowest concentration (0.0001×), approximately 2000 droplets of 215 μm in diameter (~5.2 nL) were analyzed to calculate the number of gene targets in the reservoirs. In comparison, for the highest concentration (0.1×), approximately 12,000 droplets of 47 μm diameter (~54 pL) were analyzed to calculate the number of gene targets in the reservoirs. The average DNA copy counts were determined to be 7, 63, 727, and 6175, respectively, from lowest to highest concentration (reported in Table S5). Considering these results combined, the Shigella sonnei target had a dynamic range of approximately 7–6200 copies/µL, as shown in Figure 6B with a linear relationship between the calculated DNA copy number and the dilution factor, with an R^2^ value of 0.99538. The lower limit of the dynamic range can be further improved by using larger sample volumes and better stabilizing of the large droplets. In this study, we kept our sample volume at 100 μL. For lower-concentration samples, we could further increase the sample volume to generate more droplets with larger sizes. As the droplet size increased, it became more difficult to maintain the integrity of all droplet populations during amplification. In future studies, we will explore amplification reactions that occur under milder conditions (e.g., recombinase polymerase amplification) to achieve robust detection with larger droplet sizes.

## 4. Conclusions

In summary, we have developed a compact, portable, and high dynamic range ddLAMP system that can be used for the wide detection of nucleic acids at a POC or low-resource setting. With a single vibrating sharp-tip capillary, a large range of droplet sizes is possible, making a large dynamic range possible. By changing the type of capillary, the same vibrating sharp-tip capillary mechanism can be used for a simple method of DNA fragmentation with low-volume consumption of DNA samples. We have also improved the time required for data analysis by automating the image processing and quantification of results. Because of these simple compact, and portable vibrating sharp-tip capillary devices as well as the automation of results, we have reported a high dynamic range ddLAMP system for detecting nucleic acids from approximately 2 to 6000 copies/µL under current conditions. In the present system, both the droplet generation and amplification process are compatible with a portable system. For future studies, we will investigate the possibilities of employing a portable imaging platform to achieve a fully portable digital detection system.

## Data Availability

The original contributions presented in the study are included in the article/Supplementary Material; further inquiries can be directed to the corresponding author.

## Supplementary Materials

The following supporting information can be downloaded at: https://www.mdpi.com/article/10.3390/s24134266/s1, Figure S1: Comparison of fluorescence intensity difference using different reaction time intervals; Figure S2: General overview of CellProfiler image processing; Figure S3: Linear relationship of manual image analysis with expanded view of low concentrations for Vibrio cholerae; Figure S4: Comparison of image analysis methods for Vibrio cholerae; Table S1: List of primers used; Table S2: Droplet size comparison of a 20 μm tip operating at 98.4 kHz and 3.5 Vpp; Table S3: Droplet size comparison of a 20 μm tip operating at 98.4 kHz and 4.0 Vpp; Table S4: Data summary of DNA counting methods for Vibrio cholerae; Table S5: Data summary of DNA counts at different concentrations for Shigella sonnei; Video S1: Continuous flow of liquid from vibrating sharp-tip capillary device; Video S2: Continuous flow and droplet generation from vibrating sharp-tip capillary device; Video S3: On-demand change in droplet volume.
