# Supplementary material for: Nucleic Acid Target Sensing Using a Vibrating Sharp-Tip Capillary and Digital Droplet Loop-Mediated Isothermal Amplification (ddLAMP)
Source: Sensors (Basel). 2024 Jun 30;24(13):4266. doi: 10.3390/s24134266 (PMC11243892; doi:10.3390/s24134266)
Supplement: Supplementary file 1 [file sensors-24-04266-s001.zip › sensors-3056089-supplementary.pdf]

Supporting Information

Nucleic Acid Target Sensing Using a Vibrating Sharp-Tip Capillary and Digital Droplet Loop-Mediated Isothermal Amplification (ddLAMP)

Bethany J. Fike [1], Kathrine Curtin [1,2] and Peng Li [1,*]

[1] C. Eugene Bennett Department of Chemistry, West Virginia University, Morgantown, WV 26506, USA
[2] Department of Mechanical and Aerospace Engineering, West Virginia University, Morgantown, WV 26506, USA
* Correspondence: peng.li@mail.wvu.edu

Table of contents:

Figure S1 Comparison of fluorescence intensity difference using different reaction time intervals.

Figure S2 General overview of CellProfiler image processing.

Figure S3 Linear relationship of manual image analysis with expanded view of low concentrations for Vibrio cholerae.

Figure S4 Comparison of image analysis methods for Vibrio cholerae.

Table S1 List of primers used.

Table S2 Droplet size comparison of a 20-µm tip operating at 98.4 kHz and 3.5 Vpp.

Table S3 Droplet size comparison of a 20-µm tip operating at 98.4 kHz and 4.0 Vpp.

Table S4 Data summary of DNA counting methods for Vibrio cholerae.

Table S5 Data summary of DNA counts at different concentrations for Shigella sonnei.

Video S1 Continuous flow of liquid from vibrating sharp-tip capillary device.

Video S2 Continuous flow and droplet generation from vibrating sharp-tip capillary device.

Video S3 On-demand change in droplet volume.

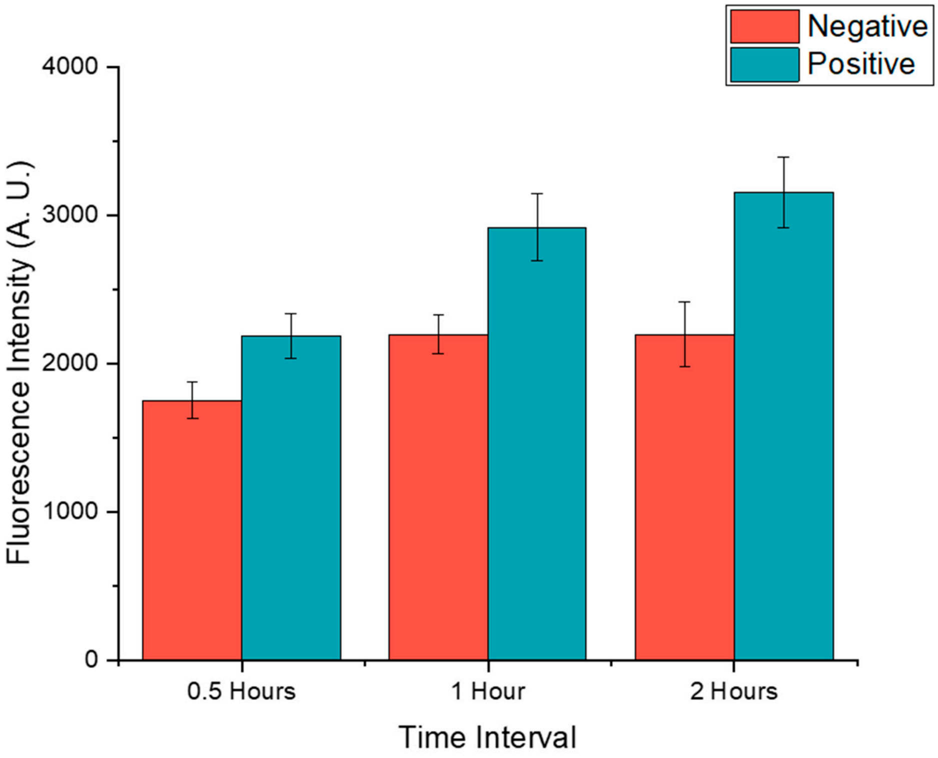

Figure S1: Comparison of fluorescence intensity difference using different reaction time intervals.

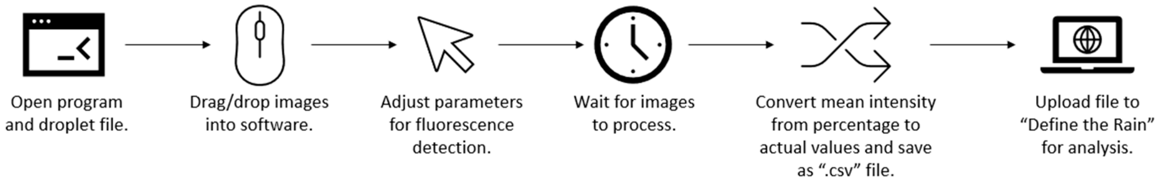

Figure S2: General overview of CellProfiler image processing.

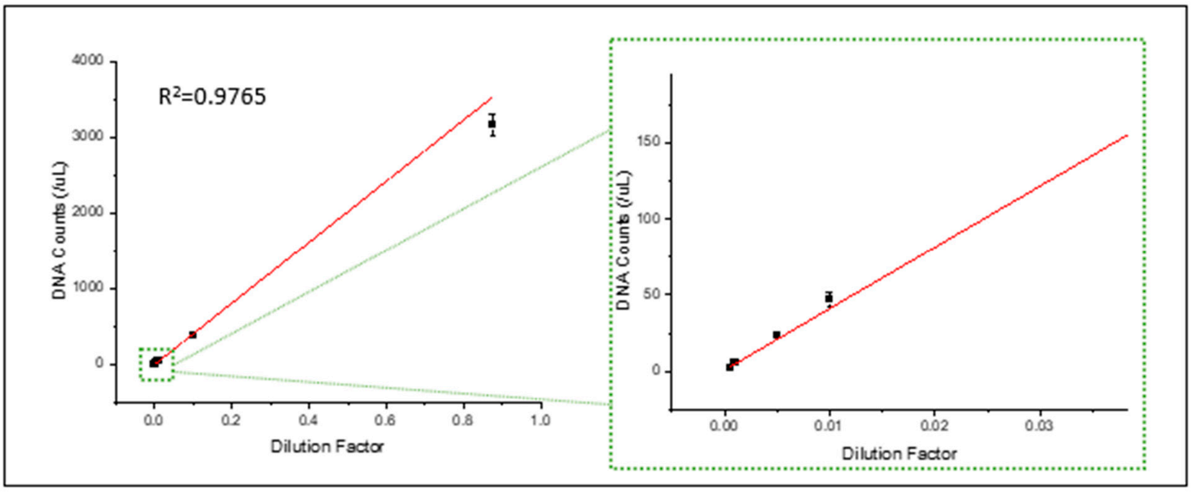

Figure S3: Linear relationship of manual image analysis with expanded view of low concentration for Vibrio cholerae.

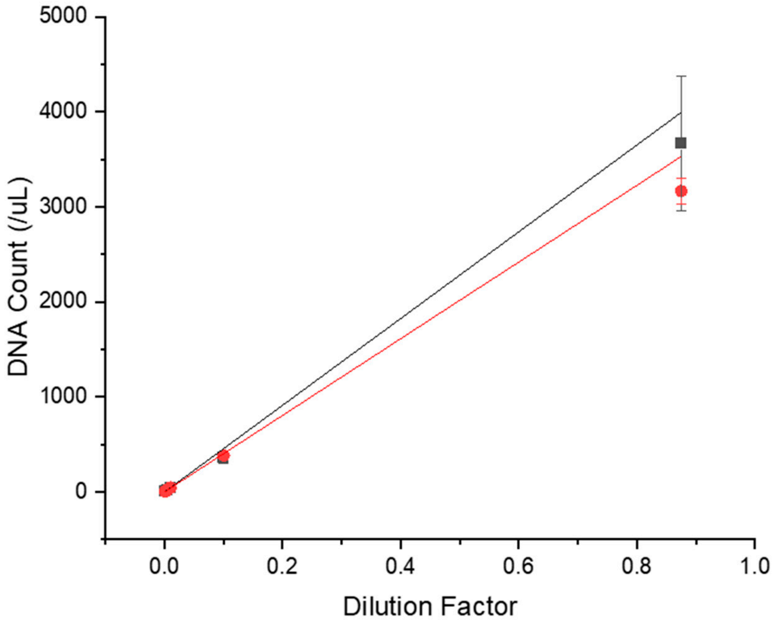

Figure S4: Comparison of image analysis methods for Vibrio cholerae. (Black square – automated image analysis; Red circle – manual image analysis)

Table S1: List of primers used.[1]

Primer Reference	Sequence (5' to 3')
Listeria Monocytogenes Loop-B	TGCATAGCCGACATGAGAGG
Listeria Monocytogenes Loop-F	CATCTTTCAAAGGCGTGGC
Listeria Monocytogenes F3	GGCAACCTGCCTGTAAGTTG
Listeria Monocytogenes B3	TGCCTCCCGTAGGAGTCT
Listeria Monocytogenes FIP	GCCCATCTGTAAGCGATAGCCGAACCGGGGCTAATACCGAA
Listeria Monocytogenes BIP	AATGGCCTACCAAGGCAACGATGTCTCAGTCCCAGTGTGG
Vibrio Cholerae Loop-B	CTTCTGTGTGGTATGCCAATAT
Vibrio Cholerae Loop-F	--
Vibrio Cholerae F3	GCTTTAATGGTACGGGTACT
Vibrio Cholerae B3	CCGTGGATTTGGCATCTG
Vibrio Cholerae FIP	TAATCAAAGCCAACGTTAGCAGCCTGGATTGAGTGATTTAAAACTTG
Vibrio Cholerae BIP	TATGCTCAATGATAGCTGGTTCCTTGTAGGTTGCCGTTGTT
Shigella Sonnei Loop-B	--
Shigella Sonnei Loop-F	CTCACAGCTCTCAGTGGC
Shigella Sonnei F3	GCTGGAAAAACTCAGTGCCT
Shigella Sonnei B3	GGAACATTTCCCTGCCCA
Shigella Sonnei FIP	CATGTGAGCGCGACACGGTCACAGCAGTCTTTCGCTGTTG
Shigella Sonnei BIP	ATCTCCGGAAAACCCTCCTGGTAGCGCCGGTATCATTATCGA

Table S2: Droplet size comparison of a 20-µm tip operating at 98.4 kHz and 3.5 Vpp.

**Pulse Frequency (Hz)**	**Droplet Radius (µm)**	**Droplet Volume (nL)**
5	142.7 ± 3.5	12.2 ± 0.9
10	112.3 ± 2.9	5.9 ± 0.5
20	88.2 ± 2.2	2.9 ± 0.2
50	71.4 ± 0.5	1.53 ± 0.03
100	55.7 ± 0.3	0.72 ± 0.01
200	42.8 ± 0.3	0.328 ± 0.006
500	28.2 ± 0.3	0.094 ± 0.003
1000	19.6 ± 0.3	0.032 ± 0.001
2000	11.3 ± 0.3	0.0061 ± 0.0005
4000	7.5 ± 0.5	0.0018 ± 0.0003
5000	5.4 ± 0.6	0.0007 ± 0.0002

Table S3: Droplet size comparison of a 20-µm tip operating at 98.4 kHz and 4.0 Vpp.

**Pulse Frequency (Hz)**	**Droplet Radius (µm)**	**Droplet Volume (nL)**
5	174.4 ± 2.3	22.2 ± 0.9
10	138.1 ± 1.3	11.0 ± 0.3
20	109.5 ± 0.5	5.50 ± 0.07
50	80.3 ± 0.7	2.17 ± 0.06
100	63.4 ± 0.4	1.07 ± 0.02
200	49.0 ± 0.3	0.494 ± 0.008
500	32.6 ± 0.4	0.145 ± 0.006
1000	23.0 ± 0.3	0.051 ± 0.002
2000	13.8 ± 0.3	0.0109 ± 0.0006
4000	9.2 ± 0.4	0.0033 ± 0.0004
5000	6.5 ± 0.4	0.0012 ± 0.0002

Table S4: Data summary of DNA counting methods for Vibrio Cholerae.

**Analysis Method**	**Dilution Factor**	**DNA Count (/µL) Reservoir 1**	**DNA Count (/µL) Reservoir 2**	**DNA Count (/µL) Reservoir 3**	**DNA Count (/µL) Reservoir 4**	**DNA Count (/µL) Average**
Fully Automated	0.0005x	2.3	2.1	2.4	2.7	2.3 ± 0.3
	0.001x	5.9	5.7	5.2	5.7	5.6 ± 0.3
	0.005x	22.7	23.6	23.7	23.0	23.3 ± 0.5
	0.01x	48.4	42.5	44.3	48.9	46.0 ± 3.1
	0.1x	425.5	349.4	279.6	384.3	359.7 ± 61.8
	0.875x	3142.2	2994.4	4460.2	4057.9	3663.7 ± 709.4
Fully Manual	0.0005x	2.6	2.3	2.5	2.8	2.5 ± 0.2
	0.001x	6.2	5.8	5.4	5.6	5.7 ± 0.3
	0.005x	22.8	25.4	23.7	23.7	23.9 ± 1.1
	0.01x	50.2	42.2	44.8	51.6	47.2 ± 4.5
	0.1x	366.5	338.3	407.4	425.3	384.4 ± 39.4
	0.875x	2976.5	3257.0	3147.4	3283.7	3166.1 ± 139.5

Table S5: Data summary of DNA counts at different concentrations for Shigella sonnei.

**Dilution Factor**	**DNA count (/µL) Reservoir 1**	**DNA count (/µL) Reservoir 2**	**DNA count (/µL) Reservoir 3**	**DNA count (/µL) Reservoir 4**	**DNA count (/µL) Average**
0.0001x	5.4	6.4	7.4	7.1	6.6 ± 0.9
0.001x	64.8	58.8	65.7	61.3	62.6 ± 3.2
0.01x	748.2	708.4	788.6	664.5	727.4 ± 53.2
0.1x	5932.6	6686.3	5767.8	6313.6	6175.1 ± 410.4

References:

1. Curtin, K.; Wang, J.; Fike, B. J.; Binkley, B.; Li, P. A 3D printed microfluidic device for scalable multiplexed CRISPR-cas12a biosensing. *Biomed. Microdevices.* **2023**, *25*, 34. DOI: 10.1007/s10544-023-00675-x.
